# Suppression of the Growth of Intermetallic Compound Layers with the Addition of Graphene Nano-Sheets to an Epoxy Sn–Ag–Cu Solder on a Cu Substrate

**DOI:** 10.3390/ma12060936

**Published:** 2019-03-21

**Authors:** Min-Soo Kang, Do-Seok Kim, Young-Eui Shin

**Affiliations:** School of Mechanical Engineering, Chung-Ang University, 84, Heukseok-ro, Dongjak-gu, Seoul 06974, Korea; kang10101@cau.ac.kr (M.-S.K.); kdoseok@naver.com (D.-S.K.)

**Keywords:** graphene nano-sheets (GNSs), epoxy solder, intermetallic compound (IMC)

## Abstract

This study investigated the suppression of the growth of the intermetallic compound (IMC) layer that forms between epoxy solder joints and the substrate in electronic packaging by adding graphene nano-sheets (GNSs) to 96.5Sn–3.0Ag–0.5Cu (wt %, SAC305) solder whose bonding characteristics had been strengthened with a polymer. IMC growth was induced in isothermal aging tests at 150 °C, 125 °C and 85 °C for 504 h (21 days). Activation energies were calculated based on the IMC layer thickness, temperature, and time. The activation energy required for the formation of IMCs was 45.5 KJ/mol for the plain epoxy solder, 52.8 KJ/mol for the 0.01%-GNS solder, 62.5 KJ/mol for the 0.05%-GNS solder, and 68.7 KJ/mol for the 0.1%-GNS solder. Thus, the preventive effects were higher for increasing concentrations of GNS in the epoxy solder. In addition, shear tests were employed on the solder joints to analyze the relationship between the addition of GNSs and the bonding characteristics of the solder joints. It was found that the addition of GNSs to epoxy solder weakened the bonding characteristics of the solder, but not critically so because the shear force was higher than for normal solder (i.e., without the addition of epoxy). Thus, the addition of a small amount of GNSs to epoxy solder can suppress the formation of an IMC layer during isothermal aging without significantly weakening the bonding characteristics of the epoxy solder paste.

## 1. Introduction

The use of lead (Pb) in the electronic packaging industry has been greatly reduced because it poses a threat to the environment and to public health. As such, worldwide environmental regulations such as the “Restriction of Hazardous Substances” (RoHS) from the European Union ban the use of dangerous and harmful substances in electronic products [1,2,3,4]. As a response to this, most electronic appliance manufacturers employ lead-free solder. Several environmentally friendly Sn–based alloys such as Sn–3.0Ag–0.5Cu [5], Sn–14Bi-5In [6], Sn–0.7Cu [7], Sn–9Zn [8], Sn–8Zn-3Bi [9], and Sn–58Bi [10] are considered the most promising candidates to replace toxic Sn–Pb alloys in electronic packaging systems. Of these, Sn–Ag–Cu alloys have drawn particular attention for their advantageous properties, such as their relatively low melting point compared with Sn–Ag binary eutectic alloys, their generally superior mechanical properties, and relatively good solderability [10,11,12,13,14].

However, Sn–Ag–Cu solder joints are prone to cracking due to the difference in the coefficients of thermal expansion (CTEs) between the copper (Cu) substrate and the solder. Another serious drawback is the formation of an intermetallic compound (IMCs) layer within the solder joint. Past research has looked to solve the issue of crack propagation in solder joints using a polymer underfill system. Recently, underfill bonding mechanisms have become particularly common in ball grid array (BGA) chip solder joints. In addition, solders containing epoxy have been developed to suppress crack propagation in solder joints. Generally, epoxy solder joints enhance the bonding strength of solder joints through mechanical locking around the solder, ensuring firm bonding between the solder and the substrate [15,16,17]. However, the growth of IMC layers at high temperatures remains a problem. A dual Cu–Sn IMCs layer consisting of Cu_6_Sn_5_ and Cu_3_Sn can form within solder joints between the Cu substrate and the solder interface. Generally, IMC growth in Sn–Ag–Cu solder joints is more rapid than in eutectic Sn–Pb solder joints due to their higher reflow temperature and significant proportion of Sn [18,19,20]. Thick intermetallic growth degrades the bonding strength due to the brittle nature of the IMC layer and the mismatch in physical properties such as the CTE and elastic modulus. Excessive thickness may also decrease the ductility and strength of the solder joint [21] and increase the electrical resistance [22]. As such, it is important to suppress the growth of the IMC layer in all kinds of solder joint.

In this study, the effect of the addition of graphene nano-sheets (GNSs) to SAC305 epoxy solder on the growth of the Cu–Sn IMC layer was investigated. The influence of the GNSs on the bonding characteristics of the solder joints was also investigated in shear tests.

## 2. Experimental

**Figure 1:**
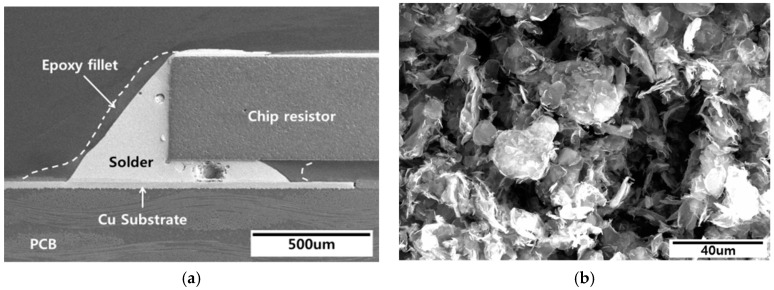
SEM images of (**a**) the structure of the specimens and (**b**) graphene nano-sheets (GNSs).

Plain epoxy solder paste was produced using SAC305 solder powder with a particle size of 10–25 µm, a flux, an amine-containing epoxy as a binder, a small amount of rosin, a surfactant, and a carboxyl-group activator. GNS epoxy solder pastes were prepared by mixing GNSs (at 0.01, 0.05, 0.1 wt %) with plain epoxy solder, with the mixture blended mechanically for 2 h at 200 rpm. Scanning electron microscopy (SEM, Hitachi S-3400N, Tokyo, Japan) images of the GNSs used in this study are shown in Figure 1b. GNSs are typically produced from natural graphite through chemical exfoliation, thermal shock and shear, or the use of a plasma reactor. In this paper, the GNSs had an average thickness of approximately 6–8 nanometers and an average particle size of 10 microns. The test specimens consisted of a chip resistor (R3216; L: 3.2 mm, W: 1.6 mm, H: 0.55 mm) soldered to a printed circuit board (PCB) with an organic solderability preservative (OSP) finish. The solder paste (plain epoxy or GNS epoxy) was screen-printed onto a Cu pad using the squeeze method, and the R3216 chip was placed on the solder paste. The solder paste was reflowed at a temperature profile of 240 °C for 100 s. An example specimen is presented in Figure 1a. It has been found that the melting point of conventional solder increases slightly if GNSs are added [23,24], while SAC305 epoxy solder also requires higher temperatures for the reflow process than does conventional SAC305 solder. Thus, in this study, the increase in the melting point with the addition of GNSs did not have a significant influence on the reflow process because the epoxy solder needed to be reflowed at a much higher temperature regardless.

**Figure 2:**
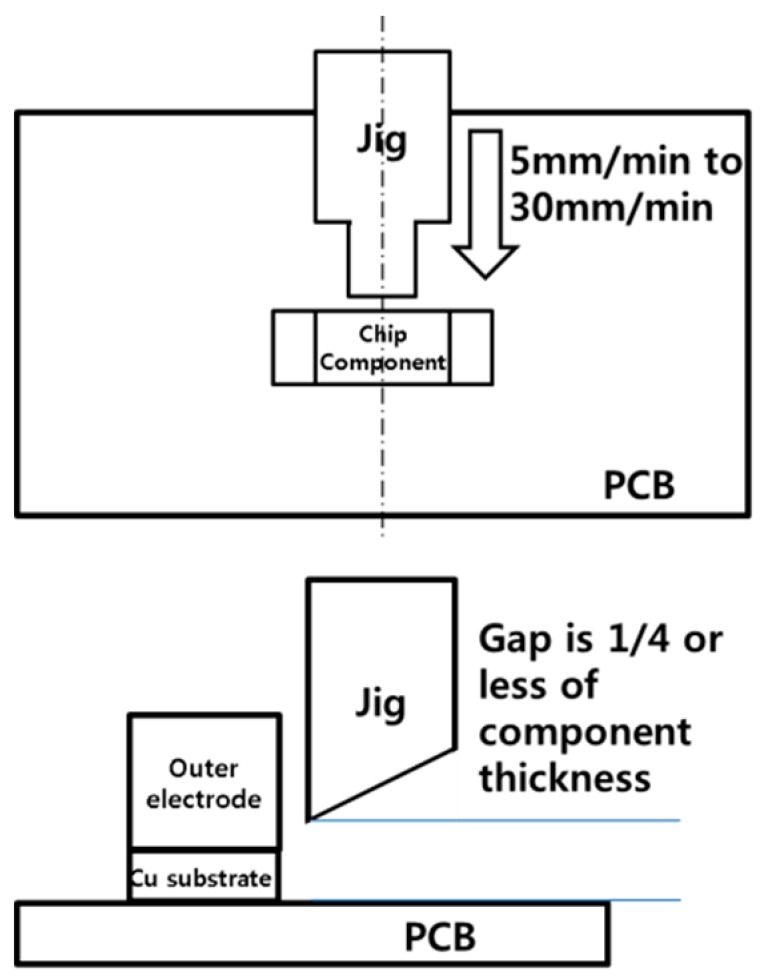
Schematic diagram of the shear testing.

Following fabrication of the specimens, the solder joints were aged in a thermal chamber at 85 °C, 125 °C and 150 °C for 21 days (504 h) following the JESD22-A103C standard [25] (i.e., high-temperature storage life). Shear forces were then measured to determine the effect of the GNSs on the bonding characteristics of the solder joints. The shear forces were tested using a JIS Z 3198-7, which operates at a shear speed of 5–30 mm/min and a height that is 25% or less of the component thickness (Figure 2). We measured shear forces at a shear speed of 10 mm/min and a height of 100 µm (0.1 mm) from the Cu substrate. Finally, the solder joints were cross-sectioned, polished, and etched for 30 s with 10 vol % hydrochloric acid in ethanol in order to observe the IMC layer in the solder joints. The thickness of the IMC layer was measured based on SEM images, with the average of the maximum and minimum thickness for each specimen used in the analysis.

## 3. Results

**Figure 3:**
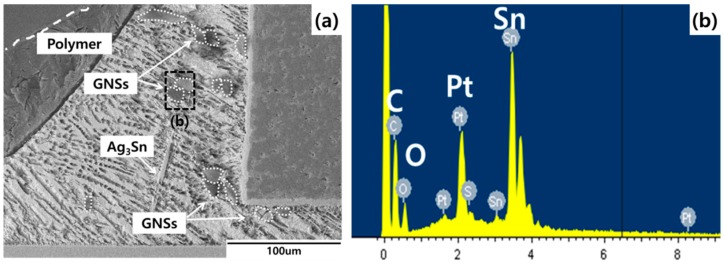
Dispersion of graphene nano-sheets(GNSs) in the solder joints: (**a**) cross-section image of a GNS epoxy solder joint and (**b**) EDS analysis results.

**Figure 4:**
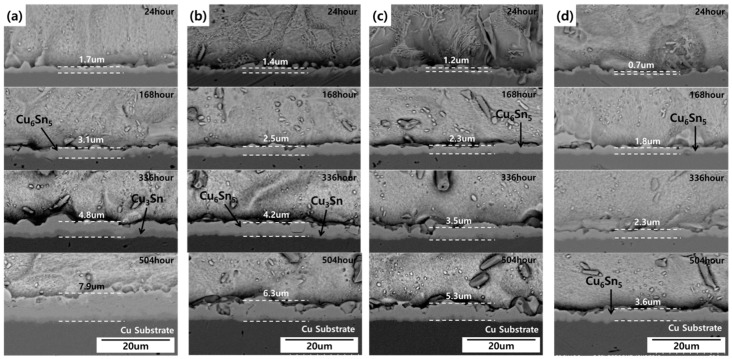
SEM images showing the growth of the IMC layer during the 150℃ aging test: (**a**) plain epoxy solder joints, (**b**) 0.01%-GNS epoxy solder joints, (**c**) 0.05%-GNS epoxy solder joints, (**d**) 0.1%-GNS epoxy solder joints.

**Figure 5:**
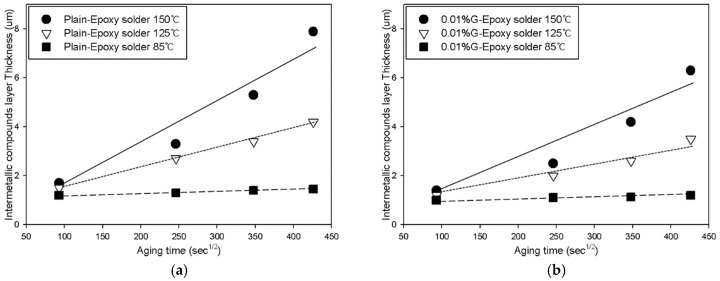

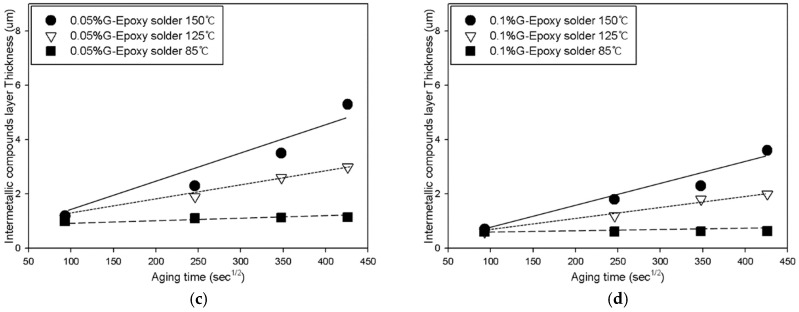
IMC layer growth over time in the solder joints: (**a**) plain solder joint; (**b**) 0.01%-GNS solder joint; (**c**) 0.05%-GNS solder joint; (**d**) IMC thickness in the 0.1%-GNS solder joint.

The SEM image in Figure 3 confirms that the GNSs were distributed throughout the solder joint, while the SEM images in Figure 4 depict the growth of the IMC layer in the solder joint. Generally, IMC layers form at the interface between the solder and a substrate, i.e., between the solder and the chip electrode and/or between the solder and the Cu substrate. In this study, the IMC layer formed at the solder/Cu substrate interface only because the chip electrode was coated with a nickel (Ni) layer. The Ni layer suppresses Sn diffusion in the solder joint, protecting the chip from damage. However, the solder/Cu substrate interface does not have a barrier that prevents Sn diffusion given that it was finished with only OSP and flux for soldering. As can be observed in Figure 4, the IMC layer grew consistently over time. Two sub-layers were observed in the solder: Cu_6_Sn_5_ adjacent to the solder matrix, which appeared from the start of the aging process, and Cu_3_Sn adjacent to the Cu substrate, which appeared after 336 h of aging. After aging at 150 °C, the thickness of the IMC layer was 7.9 µm in the plain epoxy solder joint, 6.3 µm in the 0.01%-GNS solder joint, 5.3 µm in the 0.05%-GNS solder joint, and 3.6 µm in the 0.1%-GNS solder joint. Figure 5 presents a summary of the thickness of the IMC layer over time for the three test temperatures and four solder paste formulations.

The kinetics of IMC layer formation is diffusion-controlled, depending on Sn and Cu diffusion at the Sn/Cu substrate interface as a function of time and temperature [26,27,28,29]. In general, the growth of the IMC layer should follow the square root time law, with the thickness of the layer in a diffusion couple expressed as the simple parabolic equation shown in Equation (1):

(1) $$d_{t}=d_{0}+{\left(Dt\right)}^{1/2}$$

where *dt* is the thickness of the IMC layer (µm), *d_0_* is the initial thickness of the IMC layer (µm), *D* is the growth coefficient, and *t* is the reaction time. Equation (1) can be re-written as Equation (2):

(2) $$d_{t}-d_{0}={\left(Dt\right)}^{1/2}$$

*D* is related to the diffusion coefficient of the atomic elements in the IMC layer and can be obtained from a linear regression line. The growth of an IMC layer is a diffusion-dominant process, thus, the Arrhenius relationship is applicable. The activation energy for IMC growth can be calculated using this relationship:

(3) $$D=D_{0}\exp\left(-\frac{Q}{RT}\right)$$

where *D_0_* is the diffusion constant, *Q* is the activation energy, *R* is the gas constant, and *T* is the absolute temperature.

(4) $$\ln D=\ln D_{0}-Q/RT$$

**Figure 6:**
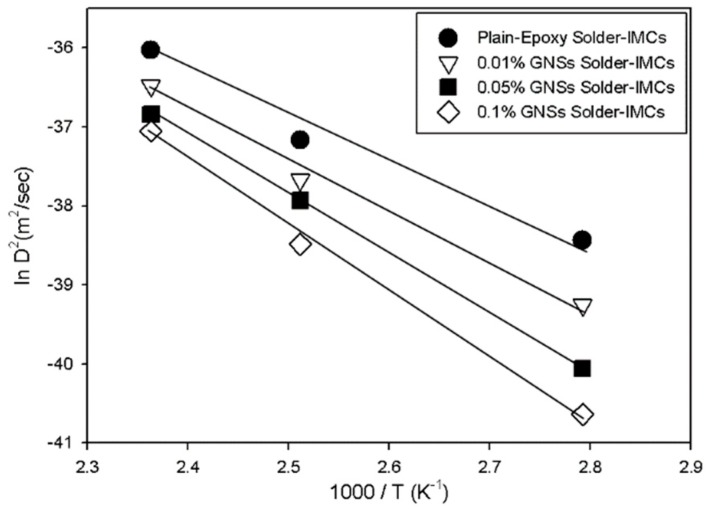
Arrhenius plot of the IMC layer for the four solder types.

The calculated Arrhenius plot is shown in Figure 6, and the activation energies were estimated from the slope of the Arrhenius plot [30,31,32]. The activation energy required to form the IMC layer in the plain epoxy solder, 0.01%-GNS solder, 0.05%-GNS solder, and 0.1%-GNS solder was 45.5 KJ/mol, 52.8 KJ/mol, 62.5 KJ/mol, and 68.7 KJ/mol, respectively. Generally, the activation energy for SAC305 solder joint/Cu substrate interfaces has been calculated at around 50.6–62.8 kJ/mol in past research [33,34]. The epoxy does not influence the activation energy in solder joints; it merely provides support for the solder joints. Thus, the activation energy of the plain epoxy solder joint in the present research was similar to conventional solder joints. In this study, the 0.1%-GNS solder joint exhibited the highest activation energy. This means that the presence of GNSs increases the amount of energy required for Sn/Cu diffusion and the subsequent formation of IMCs in GNS solder joints. The GNSs can, thus, be regarded as an effective diffusion barrier in solder joints. GNSs have a large surface area compared to their thickness, and this high specific surface area can suppress the diffusion of atoms [23,27,35]. Importantly, the GNSs do not react with or coarsen the solder matrix.

**Figure 7:**
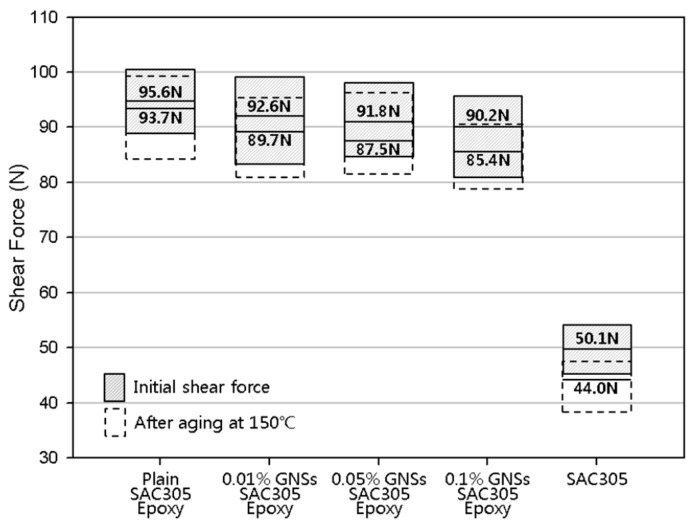
The weakening of the shear force after aging tests at 150 °C.

Shear forces were assessed to investigate the effect of GNSs on the bonding characteristics of the solder joints (Figure 7). The initial shear force was 95.6 N, 92.6 N, 91.8 N, and 90.2 N for the plain epoxy solder, 0.01%-GNS solder, 0.05%-GNS solder, and 0.1%-GNS solder, respectively. The conventional SAC305 (i.e., without epoxy) solder joint had a shear force of 50.1 N. The GNS-containing solders had a lower shear force than the plain epoxy solder, with the 0.1%-GNS formulation exhibiting the lowest force. However, the shear forces for all of the epoxy solder formulations in this study (i.e., plain, 0.01%-GNS, 0.05%-GNS, and 0.1%-GNS) were almost twice as high as conventional SAC305 solder joints.

**Figure 8:**
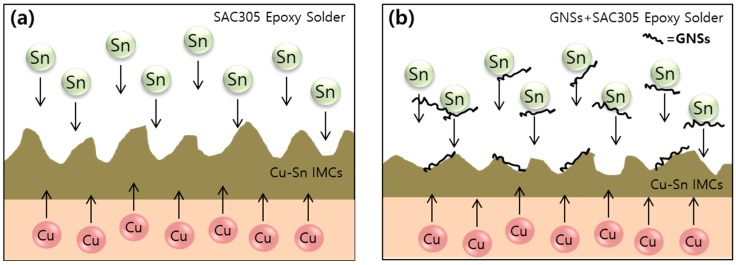
Schematic diagram of IMC growth in (**a**) SAC305 epoxy solder joints and (**b**) GNS/SAC305 epoxy solder joints.

The shear force was then compared with specimens that had been aged at 150 °C because the thickness of the IMC layers was greatest at this temperature and because the effect of GNSs in the epoxy solder was similar in every solder joint. After the aging at 150 °C, the decrease in the shear force was 2.0% for plain epoxy solder joint, 3.1% for the 0.01%-GNS solder joint, 4.7% for the 0.05%-GNS solder joint, and 5.3% for the 0.1%-GNS solder joint, while that for the conventional SAC305 solder joint was 12.2%. The addition of GNSs to the solder matrix, thus, weakens the bonding characteristics, but this is not a critical problem in solder joints because the epoxy reinforces the bonding and mechanical properties of the solder. Rather, the thickness of the IMC layer was lower in GNS solder joints because the diffusion of atoms was blocked due to the high specific surface area of the GNSs, as shown in Figure 8 [31,33]. Thus, the addition of small amounts of GNSs to epoxy solder can prevent atomic diffusion and continuously suppress IMC layer formation during isothermal aging.

## 4. Conclusions

The effect of GNSs on the growth of the IMC layer at the solder/Cu substrate interface in epoxy solder (SAC305) joints was investigated in this study. The addition of GNSs effectively suppressed this IMC layer without greatly weakening the bonding characteristics of the solder. In detail, the key results of this study were as follows:

(1) Growth of the Cu–Sn IMC layer at the solder/Cu substrate interface due to Sn and Cu atomic diffusion was confirmed with aging tests at 150 °C, 125 °C, and 85 °C. It was found that the IMC layer was suppressed by the addition GNSs to the solder. Under the harshest conditions (isothermal 150 aging for 21 days), the thickness of the IMC layer was 7.9 µm in plain epoxy solder, 6.3 µm for the 0.01%-GNS solder, 5.3 um for the 0.05%-GNS solder, and 3.6 µm for the 0.1%-GNS solder.
(2) The suppression of IMC growth was stronger with increasing GNS levels in the solder. The activation energy for the plain epoxy solder, 0.01%-GNS solder, 0.05%-GNS solder, and 0.1%-GNS solder was 45.5 KJ/mol, 52.8 KJ/mol, 62.5 KJ/mol, and 68.7 KJ/mol, respectively.
(3) The presence of GNSs in the epoxy solder paste had a slight negative effect on the bonding characteristics of the solder, but this was not critical to the function of the solder joint because the epoxy reinforced the bonding and mechanical characteristics of solder joints compared to conventional SAC305 solder joints.
